# Brain Reactivity and Vulnerability to Social Feedback Following Acute Stress in Early Adolescence

**DOI:** 10.1002/brb3.70154

**Published:** 2024-12-04

**Authors:** Zeynep Celen, Ryan J. Murray, Mariana Magnus Smith, Sondes Jouabli, Vladimira Ivanova, Eleonore Pham, Zoe Schilliger, Patrik Vuilleumier, Arnaud Merglen, Paul Klauser, Camille Piguet

**Affiliations:** ^1^ Department of Psychiatry Faculty of Medicine University of Geneva Geneva Switzerland; ^2^ Department of Neurosciences Faculty of Medicine University of Geneva Geneva Switzerland; ^3^ Division of General Pediatrics Geneva University Hospitals and Faculty of Medicine, University of Geneva Geneva Switzerland; ^4^ Centre for Psychiatric Neuroscience Department of Psychiatry Lausanne University Hospital and the University of Lausanne Lausanne Switzerland; ^5^ Service of Child and Adolescent Psychiatry, Department of Psychiatry Lausanne University Hospital and the University of Lausanne Lausanne Switzerland

**Keywords:** adolescent, feedback valence, fMRI, psychosocial stress

## Abstract

**Introduction:**

Early adolescence is a time of high psychosocial stress exposure and high stress reactivity, associated with the development of mental disorders. Understanding how the brain reacts to acute and social stressors during this period might help us detect and protect those at risk.

**Methods:**

We used functional magnetic resonance imaging to investigate acute social stress reactivity in non‐clinical adolescents between ages 13 and 15 years (*N* = 61) with a range of depression scores (Beck Depression Inventory scores 0–32). Participants underwent a modified Montreal Imaging Stress Task (MIST) with psychosocial stress condition consisting of two parts: acute stress (challenging maths) followed by social feedback (positive or negative), separated by brief recovery periods. The test condition was compared to a non‐stressful control. We examined brain responses to social feedback relative to the acute stressor and feedback valence.

**Results:**

Psychosocial stress produced differential activation in the paracingulate gyrus, insula, and deactivation in the ventral striatum. Receiving social feedback, compared to acute stress, activated cortical midline regions such as the medial prefrontal cortex and posterior cingulate cortex. Positive feedback increased activity in frontal pole and middle frontal gyrus whereas negative feedback did not show any differential response in the whole group. However, participants with depressive symptoms reacted with higher activation in the posterior cingulate cortex to negative feedback.

**Conclusion:**

We show that social feedback after an acute stressor activates regions involved in self‐referential processing, with positive feedback eliciting generally higher activation and negative feedback impacting only individuals with vulnerable mood traits during early adolescence.

## Graphical abstract

Following acute stress, in young adolescents, brain activity in motivation, and attention‐related regions are observed during positive feedback. Whereas during negative feedback, activity in self‐referential region is observed in those with depressive symptoms.

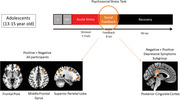

## 1 Introduction

Adolescence is a developmental period with many physical and physiological changes (Casey, Duhoux, and Cohen 2010). Increased and prolonged plasticity in several brain regions creates high vulnerability to the effects of stress exposure (Tottenham and Galván 2016). During this phase of development, the reactivity to salient environmental cues (for review: Somerville, Jones, and Casey 2010) and acute stress (Dahl and Gunnar 2009; Stroud et al. 2009) is generally increased. In addition, there is a heightened exposure to social stress relative to childhood (Spear 2000). These characteristics progressively attenuate with age, as the instability of emotional states, more pronounced during early adolescence (EA), decreases in late adolescence with the concurring development of cognitive control regions such as the prefrontal cortex (PFC) (Larson et al. 2002). However, this period may have an enduring influence on health as epidemiological studies indicate that one‐third of all lifelong mental illnesses begin by the age of 14, and more than half by the age of 24 (Solmi et al. 2022).

Research on the neural correlates of acute stress reactivity and the effects of psychosocial stress in young adolescents is still in its infancy. Acute stress results from physical or psychological events that may last for a short period of time, but can induce persistent increases in cardiovascular response and negative emotions (Feldman et al. 1999). Adaptive recovery from acute stress is linked to positive affective traits (Bostock et al. 2011). In adolescents, failure of acute stress response systems might be associated with self‐harming or suicidal behavior (Miller and Prinstein 2019). At the neural level, acute psychosocial stress in adolescents reduces responses to positive experiences in prefrontal cortical areas and insula, and blunts reward‐related activity in the striatum (Lincoln et al. 2019). A similar effect is seen with acute physical stress, such as cold stressor, which reduces ventral striatal activity (Burani et al. 2021). Psychobiological and behavioral components of the stress response in late adolescence/young adults are known to be regulated by several brain regions in the PFC and inferior parietal lobule (Wheelock et al. 2016), regions that terminate their maturation during the second decade of life (Sydnor et al. 2021).

Psychosocial stress stemming from social interaction and evaluation, especially involving peers, is a frequent event for adolescents. This is a period when the influence of peers exceeds that from family (Barnes et al. 2007), where peer relationships become complex, salient, and important (Brown and Larson 2009). Adolescents are sensitive to negative feedback in general (Heffer and Willoughby 2020) and young adults with depressive symptoms recall negative social feedback more (Xie et al. 2022). Increased reactivity to social and emotional cues is a notable feature of adolescence (Casey et al. 2019), as seen in increased PFC activity to social evaluation (Somerville et al. 2013). Altered neural processing of social feedback, either a blunted response to reward or increased reactivity to exclusion, might in turn be related to vulnerability to depression (Kujawa and Burkhouse 2017).

Some studies that focus on social feedback show that receiving either negative or positive social evaluation activates regions of the salience network, such as dorsal anterior cingulate cortex (dACC) and anterior insula (AI), irrespective of valence (Dalgleish et al. 2017). Perini et al. (2018) showed that dACC and AI are related to the salience of being judged by others, rather than social pain. Negative peer feedback processing has been associated with activation in medial PFC, amygdala, and striatum, as well as dACC and AI (Rappaport and Barch 2020). Some of these responses might reflect a dispositional risk of depression (Pagliaccio et al. 2023).

Recently, more attention has been directed toward positive emotions and the effects of positive valence. Receiving positive feedback results in higher activation in the medial and ventrolateral PFC (Davis et al. 2023). Thinking about positive memories safeguards against the detrimental effects of acute stressors and enhances mood (Speer and Delgado 2017). Positive feedback, when compared to negative, results in better learning in adolescents and especially in children, when compared to adults (van Duijvenvoorde et al. 2008; Zhuang, Feng, and Liao 2017). Ventral striatum activity after a correct response peaks in adolescence compared to childhood and adulthood (Satterthwaite et al. 2012). Even though EA might be a turning point toward learning more from negative feedback (Peters et al. 2014; van Duijvenvoorde et al. 2008) and subregions of striatal activation during negative feedback begin to correlate with learning performance in late adolescence (Peters and Crone 2017), neural reactivity to positive feedback remains a relatively understudied area of research.

The goal of our study was to investigate the brain reactivity toward social feedback after an acute stressor and identify neural markers of potential vulnerability to maladaptive stress regulation. We adopted a modified MIST previously used by our group with adults (Murray et al. 2021, 2022) to test the effects of acute stress, social feedback, and feedback valence, more specifically in adolescents. We hypothesize that test conditions should recruit brain regions related to salience, attention, and emotion. In addition, social feedback in itself, relative to the preceding stress event, should produce brain responses specific to reacting to psychosocial information while the valence of social feedback should elicit differential effects. Specifically, negative feedback may activate regions similar to those previously reported for social feedback in adolescents (Corr et al. 2020) whereas positive feedback might engage circuits of positive emotions, even after exposure to an acute stressor, an issue not previously explored in this age period. Moreover, we ask whether there is a relationship between brain activity patterns to feedback valence and depression symptoms, as vulnerability to depression is increased in adolescence (Platt, Kadosh, and Lau 2013), especially from age 15 onwards (Hankin et al. 1998). Altogether, our results shed light on factors that may help detect individual vulnerabilities and design appropriate socio‐behavioral and therapeutic approaches toward youth who undergo a challenging developmental period.

## 2 Materials and Methods

### 2.1 Participants

A total of 70 adolescents between the ages 13 and 15 years were recruited as a part of a larger study, Mindfulteen (Piguet et al. 2022). Inclusion criteria were chosen to have a large variability regarding anxiety range, therefore included subjects presenting anxiety disorders without comorbidities or remitted depressive episodes. Informed consent was obtained from participants and parents when necessary. The ethical protocol was approved by the Geneva Regional Research Ethical Committee (CCER 2019‐01731). One participant dropped out, another could not be scanned; out of the 68 scanned participants, 7 had to be excluded due to MRI recording issues (Supporting Information Material), hence we report the results of 61 subjects (35 females and 26 males).

Participants completed the State‐Trait Anxiety Inventory Scale for Children (STAI‐C) (Spielberger 1983; Spielberger et al. 1973) for trait and state anxiety and the Beck Depression Inventory (BDI) (Beck and Beamesderfer 1974) to measure depressive symptoms. Sample demographics are in Table 1. In addition, due to the wide range of BDI scores, the sample was divided, in an exploratory manner, in function of their depressive symptoms. For the BDI, we used the conventional cut‐off score of 9, assigning them into two groups: “no depressive symptoms” (BDI < 10) and “depressive symptoms” (BDI ≥ 10) (Beck, Steer, and Carbin 1988). Group differences in task performance and self‐reports were analyzed using Welch's two‐sample *t*‐test on R version 4.1.0 (R Core Team 2021).

**TABLE 1:** The sample demographics of the participants in the fMRI analysis.

*N* = 61(35 F, 26 M)	Mean	SD	Range
Age (years)	13.84	0.86	13–15
STAI‐C state	29.71	4.59	20–49
STAI‐C srait	36.24	7.17	23–50
BDI	9.52	7.31	0–32
Education grade	(80% middle school; 20% post‐mid school)		
Parental work status			
Paternal	57.4% executive professional, 18% office worker, 16.4% other, 3.3% unemployed, 4.9% n.a.		
Maternal	62.3% executive professional, 21.3% office worker, 14.8% other, 2% unemployed, 1.6% n.a.		
Ethnicity	75% Caucasian		
Puberty	(47 yes/14 no)		
Handedness	(52 right/9 left)		
Actively doing sports	(51 yes/10 no)		
Health issues	(53 none/8 yes)		
Medication	(57 none/4 yes)		
Substance/alcohol dependence	(61 none)		
Social phobia	(5 yes/13 SC/43 none)		
General anxiety	(7 yes/2 SC/52 none)		
Separation anxiety	(2 in the past/59 none)		
Major depressive episode	(11 in the past/50 none)		
Severity	(3 SC/3 light/5 moderate)		

*Note*: Possible max STAI‐C score 60, possible max BDI score 63.

Abbreviation: BDI, Beck Depression Inventory; n.a., data not available; SC, subclinical; SD, standard deviation; STAI‐C, State Trait Anxiety Scale for Children;

### 2.2 MRI Task

**FIGURE 1:**
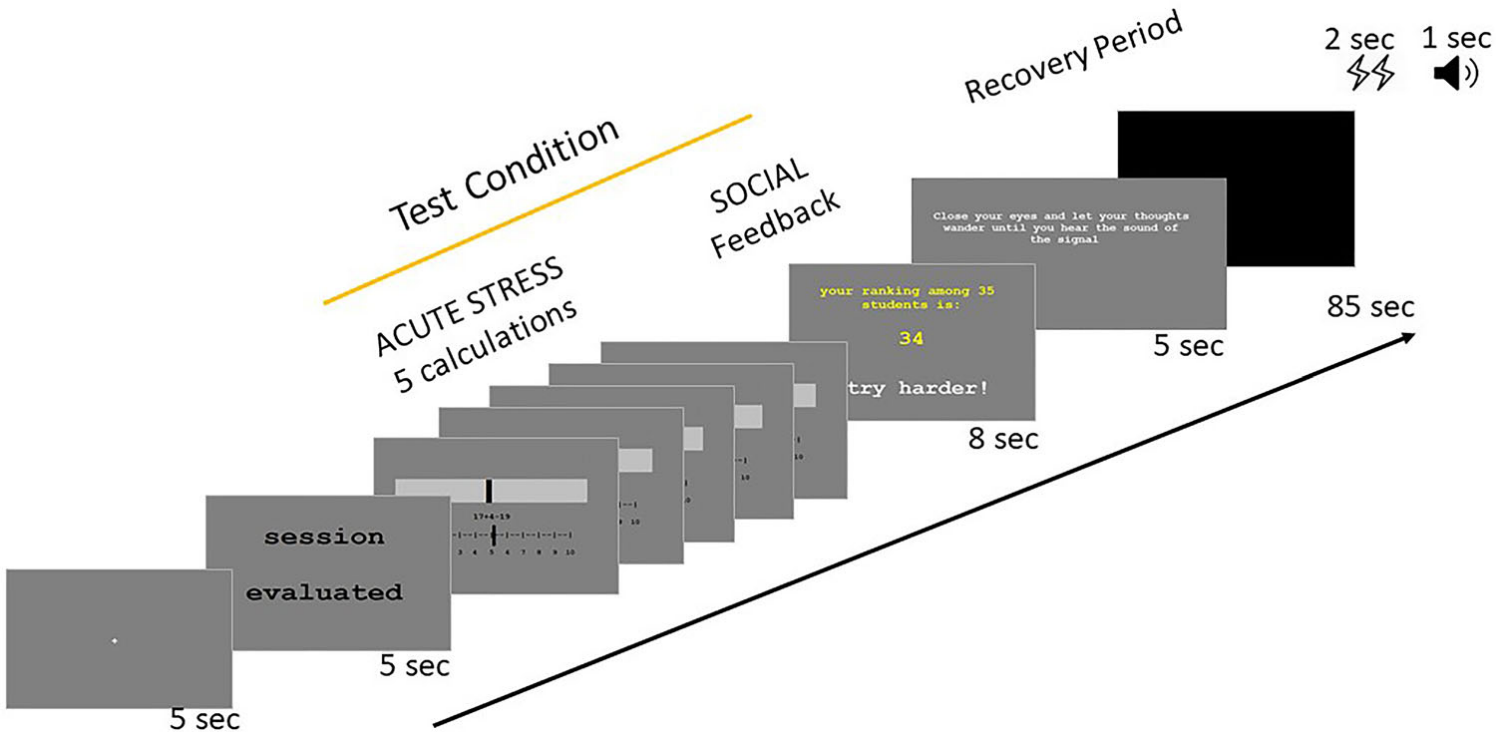
This is an adapted version of the MIST with the test condition consisting of acute stress (five challenging mental calculations) and social feedback presented consecutively. The feedback screen includes a social ranking (low ranking as in figure with a high number or high ranking for positive runs with a low number) and social feedback (negative as in figure or positive). Each condition is preceded by an information screen signifying whether performance will be evaluated (test) or not (control), followed by a 90‐s recovery period.

We updated the modified MIST previously used by our group (Murray et al. 2021). The task consisted of two runs with six trials in each run and lasted around 25 min. There were eight test trials with four positive and four negative feedbacks and four control trials with neutral feedback, pseudorandomized into six lists to counterbalance the order of conditions. Each trial began with a jittered fixation cross, followed by a 5‐s information screen signifying whether the trial is evaluated (test) or not‐evaluated (control). Test trials continued with an acute stressor of five time‐restricted, age‐appropriate challenging mental calculation questions. Participants answered with a button box, by moving the cursor freely from 0 to 10 and confirming their answer. The calculations were followed by a feedback screen for 8 s that displayed a social ranking (“your ranking among 35 students is:”); followed by a low number (high‐performance ranking) for positive feedback and a high number (low‐performance ranking) for negative feedback (Figure 1). Control trials had a series of five simple calculations with the same limited time bar, followed by a neutral feedback with no social ranking. All trials were followed by a 90‐s resting recovery period.

Before entering the scanner, participants practiced the task using the keyboard on a computer, answering simple calculations under time limitations and receiving neutral feedback. They were instructed that the task would be harder than the practice, and that it would include evaluated runs where their accuracy and speed would be compared against a group of peers.

### 2.3 Behavior

We collected calculation accuracy and response time data for each trial using EPRIME and analyzed using paired *t*‐tests using R version 4.1.0 and R studio (R Studio Team, 2021).

Participants filled in questionnaires, before entering the scanner and after exiting the scanner, reporting their stress level before, during, and after the task. The answers were given on a Likert scale from 0 to 5 (0: calm; 5: stressed). Data were analyzed using linear mixed models with the subject as a random factor. Group differences in task performance and self‐reports were analyzed using Welch's two‐sample *t*‐test.

### 2.4 Physiology

Heart rate (HR) during fMRI was recorded using an MRI‐compatible pulse oximeter, with Biopac Systems (Santa Barbara, CA). Data were analyzed on Biopac Systems Acknowledge Software (Biopac Systems, version 4.4; Biopac Systems Inc., Goleta, CA) and transformed using FIR High Pass filter fixed at 0.5 Hz. HR was calculated between a window of 40–180 BPM with 1%–5% of the peak noise rejection. Due to hand movement in the scanner and poor recording, we only retained data from 44 subjects that were of quality for analysis. A 90‐s resting period HR was analyzed by splitting into three 30‐s time bins to observe the change over time. Linear mixed models with subject as a random variable was used to look at the main effects of condition, time and their interaction, group differences were analyzed with two‐sample *t*‐test.

### 2.5 MRI Data Acquisition and Analysis

MRI data acquisition, image preprocessing and first‐level analysis are summarized in Supporting Information Material.

#### 2.5.1 Group‐Level Analysis

First, the test condition was compared to the control (acute stress and social feedback against the control calculation and neutral feedback). In addition, the two successive parts of the test condition were compared to each other: social stress (social feedback) and acute stress (challenging maths). These contrast analyses resulted in strong activations and are reported with FWE < 0.05 cluster‐forming threshold with an extent threshold *k* > 10.

Valence of social feedback was analyzed by comparing positive and negative feedback. All feedback analyses were performed after applying an exclusive incongruency mask to account for any confounding effect due to a possible sense of mismatch between calculation performance and feedback received after each set of calculations (see Murray et al. 2021). These results are FWE < 0.05 corrected at the cluster level, with a standard uncorrected cluster‐forming threshold of *p* < 0.001 (Woo, Krishnan, and Wager 2014). For more details please refer to Supporting Information Material Methods.

Analyses were conducted with SPM12 (https://www.fil.ion.ucl.ac.uk/spm/). Brain images were rendered using MRIcroGL (www.nitrc.org/projects/mricron). MNI coordinates of peaks were labelled using Harvard–Oxford cortical and subcortical maps of FSLeyes (McCarthy 2023).

#### 2.5.2 Covariate Analysis and Two‐Group Analysis

We correlated brain activity during feedback valence (Positive FB > Negative FB and Negative FB > Positive FB) with clinical scores (STAI state, STAI trait, and BDI) conducting covariate analysis. To investigate group differences, two‐sample *t*‐tests were used on SPM 12, with the contrasts Positive FB > Acute Stress and Negative FB > Acute Stress and Positive FB > Negative FB.

## 3 Results

### 3.1 Behavior

Accuracy was lower (*M* = 63.48%) and reaction time longer (*M* = 6102.4 ms) during test calculations compared to control (*M* = 95.74%; *M* = 2847.02 ms; paired samples *t*‐test, accuracy: *t*(60) = −15.47, *p* < 0.001; speed: *t*(60) = 29.84, *p* < 0.001), validating the challenging nature of these conditions. Participants in the depressive symptoms group scored significantly lower accuracy during test calculations than the no symptoms group (*M*_depsym_ = 57.27, *M*_nosym_ = 66.99; *t*(42) = −2.18, *p* = 0.03), however, there were no group differences in reaction time or in the control calculation performance.

**FIGURE 2:**
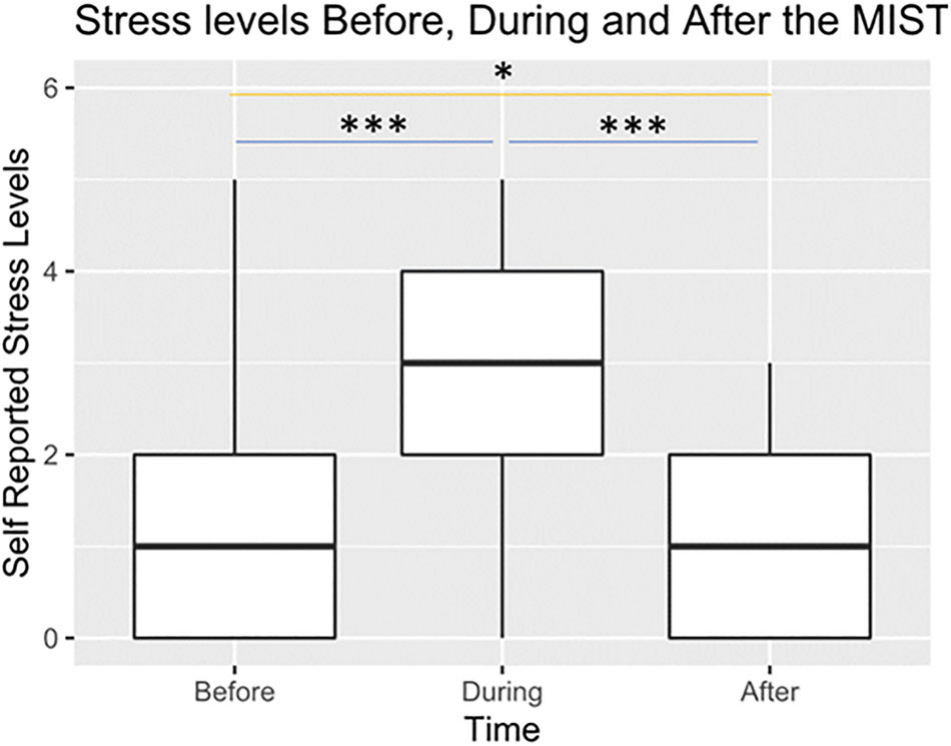
Participants reported their stress levels before entering the scanner, during the task (reported after leaving the scanner) and after leaving the scanner. Answers were given on a Likert scale (0 = *calm*; 5 =  *stressed*). Participants reported significantly more stress during the task compared to before and after (*p* < 0.001).

Self‐reported stress levels were significantly affected by time (*p* < 0.001): participants reported more stress during the task (post‐MRI questionnaire) (*β* = 1.393, SE = 0.157, *t*(120) = −8.85, *p* < 0.001) (Figure 2) and lower stress levels before (*β* = 0.344, SE = 0.157, *t*(120) = 2.19, *p* = 0.03) and after the MRI (*β* = 1.738, SE = 0.157, *t*(120) = 11.04, *p* < 0.001). There was a trend, but no significant difference between groups regarding self‐reported stress before (*t*(38) = 1.73, *p* = 0.09; *M*_depsym_ = 1.59, *M*_nosym_ = 1.03) and during (*t*(51) = 1.85, *p* = 0.07; *M*_depsym_ = 3.0, *M*_nosym_ = 2.4) the task, and no trend or significant difference after the task (*t*(35) = 0.63, *p* = 0.53; *M*_depsym_ = 1.0, *M*_nosym_ = 0.82).

### 3.2 Physiology

To assess changes in autonomic state during the recovery periods, we analyzed the mean HR within 30‐s intervals during the 90‐s rest period after each condition. There was a significant interaction of time and condition (*p* = 0.002). Follow‐up pairwise contrasts showed that both valences of the test conditions elicited a higher HR than the control condition in the first 30 s. There was no main effect of condition, but a main effect of time reflecting a sharp decrease in HR between 30 and 60 s and no further change within the last 30 s (30–60 s: ***p < 0.001; 30–90 s: ***p < 0.001; 60–90 s: *p* = 0.18), indicating sufficiency of the recovery period. There were no significant differences in HR between the two valences nor were there any group differences in mean HR during the recovery period (Figure S1).

### 3.3 Neuroimaging Results

#### 3.3.1 Psychosocial Stress

The test conditions compared to control revealed activation clusters in the left middle frontal gyrus, left IFG, paracingulate gyrus, and bilateral insula while it produced deactivation in nucleus accumbens and left supramarginal gyrus. For figure and full list of coordinates, please refer to Supporting Information Material (Figure S2; Table S2).

**FIGURE 3:**
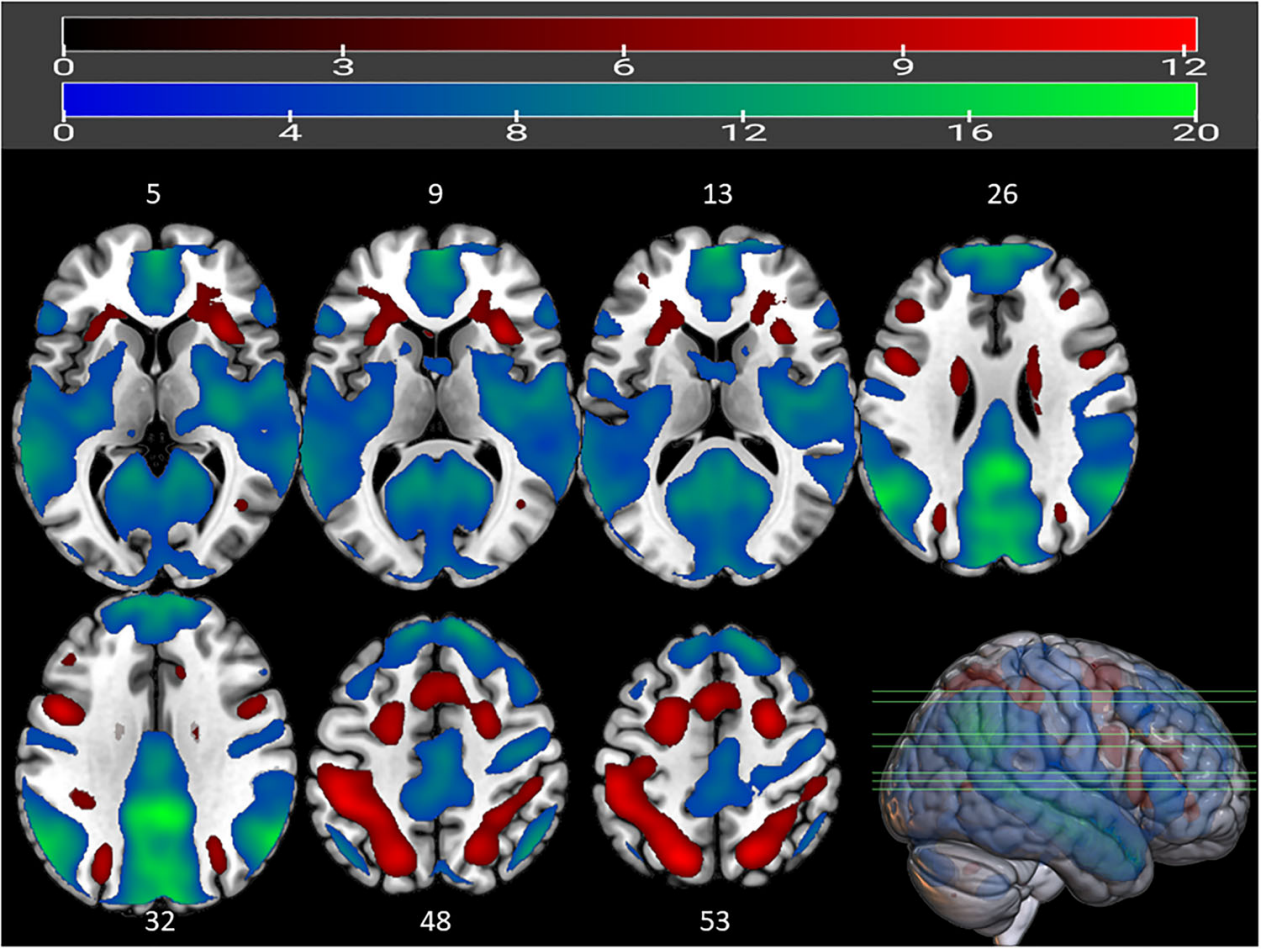
Whole‐brain analysis of BOLD signal during social feedback (blue) and acute stress math task (red) within the test condition. Red represents clusters active during acute stress (calculation) compared to the presentation of (positive or negative) feedback about performance. Regions such as insula, superior parietal gyrus, angular gyrus, and middle frontal gyrus are more active during acute stress (red clusters). Midline structures such as medial superior frontal gyrus and posterior cingulate gyrus are active during social feedback (blue green clusters). For full list of significant clusters and coordinates, see Supporting Information Material (Table S3). Cluster‐forming threshold of FWE < 0.05 with *k* > 10; bar above shows *t*‐values; *z*‐axis coordinates are noted on top of the respective axial brain slice.

Within the test condition (Figure 3), the delivery of social feedback (Positive FB and Negative FB) relative to the stressful math task itself, increased activity in midline brain structures including posterior cingulate gyrus (PCC) and medial prefrontal cortex (MPFC), as well as in bilateral angular gyrus and parts of the cerebellum. The acute stress math task, on the other hand, activated the bilateral superior parietal lobule, right superior and middle frontal gyri, as well as the bilateral insula, relative to social feedback (Table S3).

#### 3.3.2 Valence of Feedback

**FIGURE 4:**
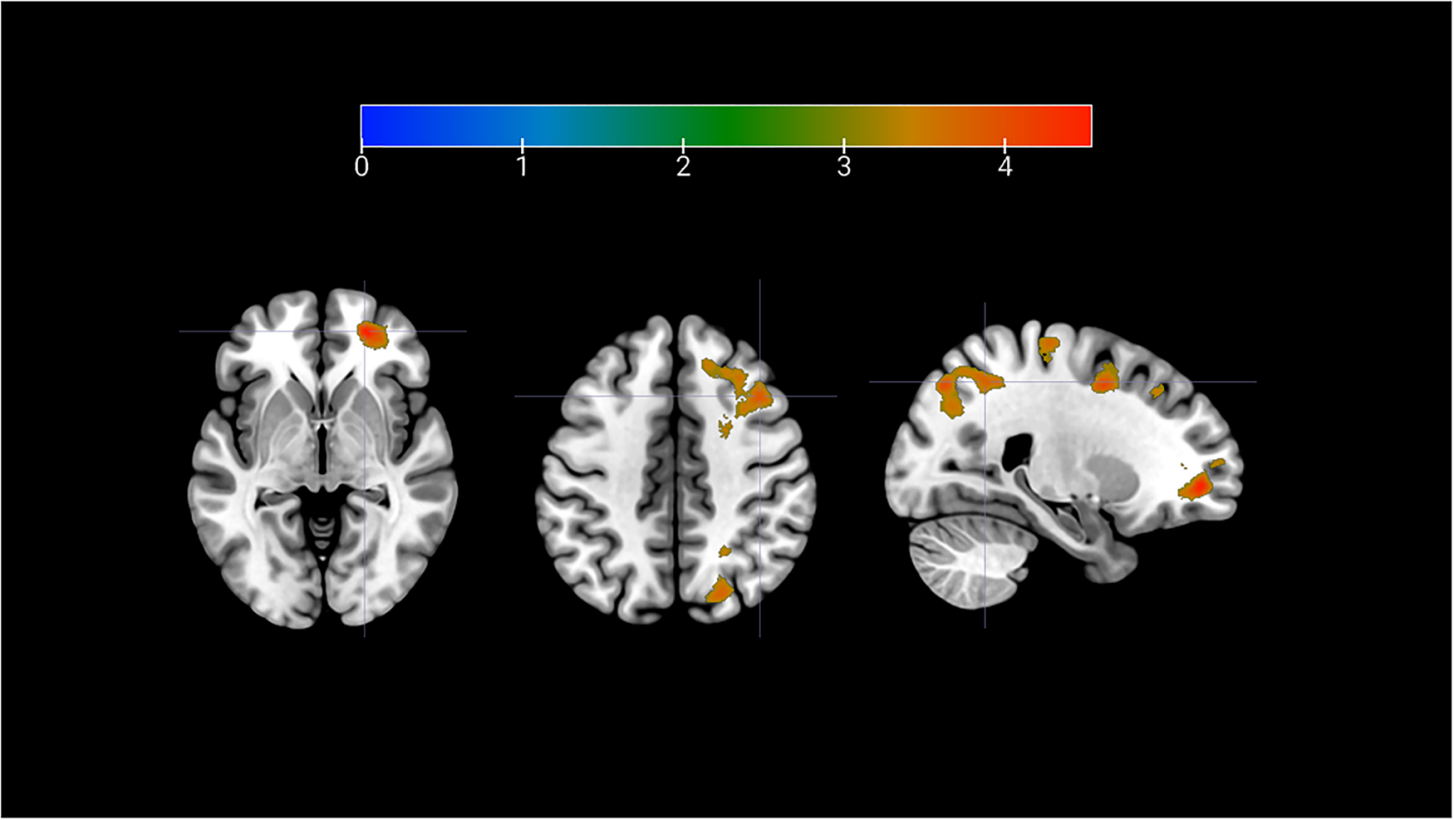
Whole brain analysis of BOLD signal during positive feedback in the all participants (Positive FB > Negative FB). Peaks indicated in figure from left to right: frontal pole (*z*‐axis = −3), middle frontal gyrus (*z*‐axis = 43) and superior parietal lobule (*x*‐axis = 24). Cluster‐forming threshold *p* < 0.001 uncorrected, cluster corrected at FWE < 0.05. For full list of activations, please refer to Table 2. Upper bar represents *T* values.

Positive feedback (Positive FB > Negative FB) increased activity in the right frontal pole, right precentral gyrus, right superior parietal lobule, right superior, and middle frontal gyrus, plus bilateral precentral gyrus (Figure 4, for list of coordinates: Table 2). Negative feedback, on the other hand, yielded no significant activity relative to positive feedback.

**TABLE 2:** Cluster activations and peaks during valence of social feedback.

				MNI coordinates		
Brain region	Side	*k*	*T*	*x*	*y*	*z*
Positive FB > Negative FB (five clusters)						
Frontal pole	R	4544	4.58	22	51	−3
Frontal pole	R		4.31	29	51	1
Frontal pole	R		4.26	35	56	8
Precentral gyrus	R	2416	4.53	17	−24	68
Precentral gyrus	R		3.87	18	−26	59
Precentral gyrus	R		3.71	13	−30	64
Superior frontal gyrus	R	4253	4.43	23	3	46
Middle frontal gyrus	R		4	40	18	43
Middle frontal gyrus	R		3.88	30	2	57
Lateral occipital cortex (LOC)—superior division	R	4357	4.4	19	−73	50
Precuneous/LOC	R		4.09	11	−68	53
Superior parietal lobule	R		3.9	24	−55	48
Precentral gyrus	L	1708	4.38	−19	−31	57
Precentral/postcentral gyrus	L		3.94	−12	−34	60
Postcentral gyrus	L		3.89	−22	−32	69
Negative FB > Positive FB (no clusters)						

*Note*: All results have a cluster‐forming threshold of *p* < 0.001 and are FWE < 0.05 corrected at the cluster level. An exclusive incongruence mask (for coordinates: Table S1) is used to account for possible sense of incongruency between performance and the predetermined feedback received.

#### 3.3.3 Valence of Feedback and Depressive Symptoms

**FIGURE 5:**
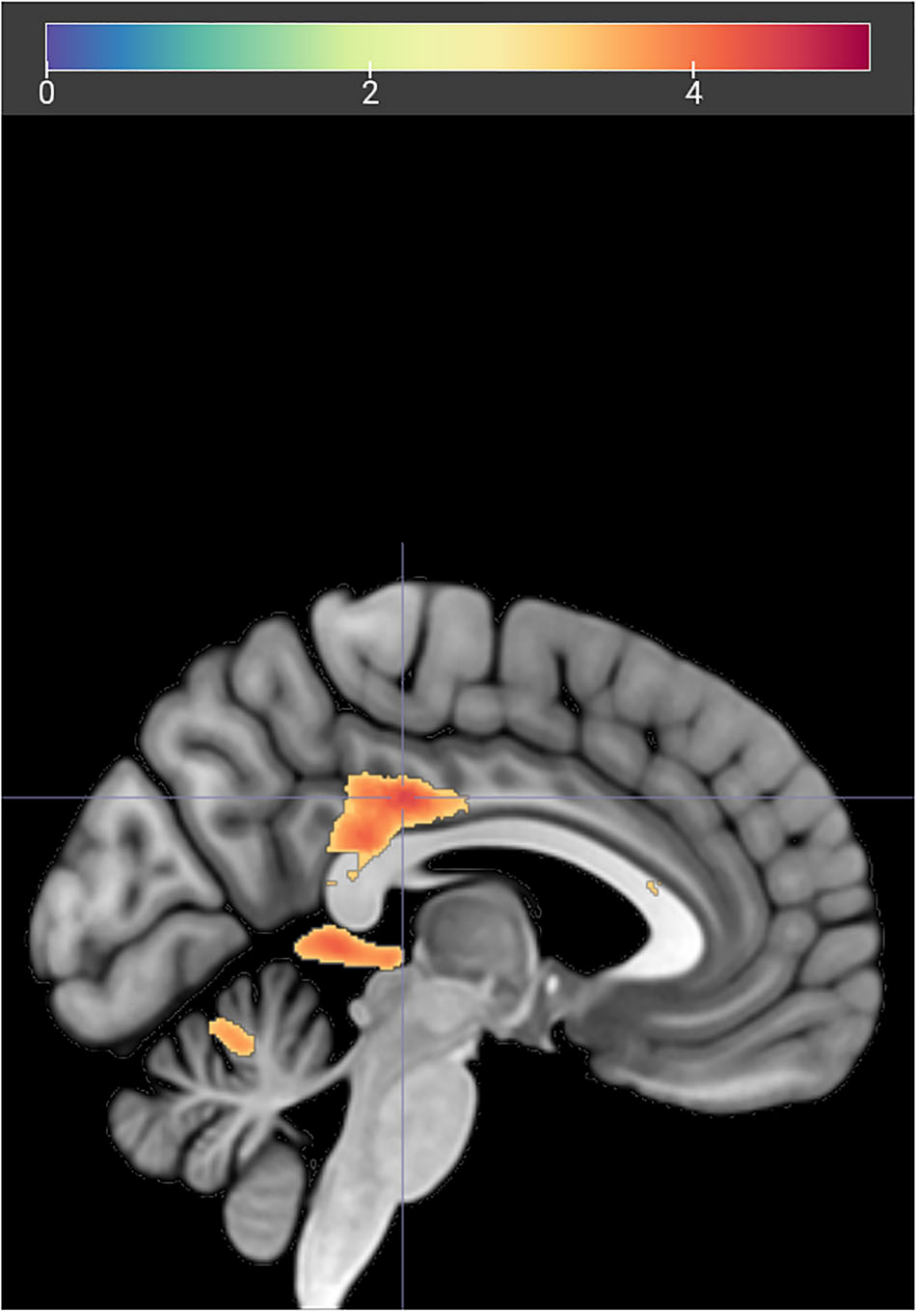
PCC activation during negative feedback, in participants with depressive symptoms. Two group *t*‐test between the groups (Depressive Symptoms > No Symptoms), comparing the contrast (Negative FB > Positive FB). Cluster‐forming threshold uncorrected *p* < 0.001, cluster corrected at FWE < 0.05. Upper bar represents *T* values.

As our participants showed a diverse range of depression scores across the whole group, we split them into two groups where 39 (19 F) had BDI scores lower than 10, that is, “no depressive symptoms” (BDI < 10). The remaining 22 participants (16 F) were assigned to the group of “depressive symptoms” with their BDI scores ranging from 10 to 32 (possible maximum 63) (Beck, Steer, and Carbin 1988). A whole brain analysis of the contrast Positive FB > Negative FB showed no significant difference between groups, whereas negative compared to positive feedback produced significant increases in several clusters for the group with depressive symptoms, with the largest effect observed in the posterior cingulate cortex (Figure 5; Table S4). We found no differences between groups in how they respond to social feedback compared to acute stress in either valance (Table S5).

## 4 Discussion

The current study examined the neural correlates of acute psychosocial stress in EA, using an adapted MIST. We found adolescents recruit attention and salience‐related regions during the cognitively challenging, stressful phase of the task itself, including insula and dorsolateral superior frontal gyrus, while they activate anterior–posterior midline structures when receiving social feedback about their performance, including MPFC and PCC. Furthermore, social feedback produced differential brain responses according to both the valence of feedback and individual psychological characteristics. Positive feedback after acute stress activated right prefrontal areas including frontal pole and middle frontal gyrus, across all participants regardless of mood, while negative feedback elicited stronger activity in posterior cingulate cortex only in participants with higher depression scores.

Brain regions most commonly activated in various stress‐induction paradigms are the bilateral insula and the inferior frontal gyrus (for review Berretz et al. 2021). This has been observed for both physiological and psychological stressors (Kogler et al. 2015). Likewise, we found that our test condition compared to the control condition activated both insula and left IFG. The latter region has also been linked to working memory and semantic processing (Liakakis, Nickel, and Seitz 2011), which are components engaged by the calculation task. In addition, we found a strong deactivation of the ventral striatum that might accord with the stressful nature of the test condition, reflecting reduced signals of motivation and reward. Psychosocial stressors that demand goal‐directed behavior and emotion regulation have already been linked to deactivation of the ventral striatum (Kogler et al. 2015), and reward‐related responses in adolescents are blunted during stress (Lincoln et al. 2019). Together with the significant self‐reported stress levels and increased HR during the evaluated condition, these results confirm that our modified MIST version functions as an effective stressor within this age group.

Evaluative social feedback (when compared to the preceding acute stressor of time‐limited math calculations) recruited the medial PFC, angular gyri, and PCC—areas central for self‐reflective processing (Johnson et al. 2002). These regions are known to be part of the default mode network, typically engaged when individuals focus on internally directed tasks, including social inferences considering the perspective and mental states of others (Buckner, Andrews‐Hanna, and Schacter 2008; Thornton, Weaverdyck, and Tamir 2019). Moreover, the MPFC in adolescents encodes social value (Kumar et al. 2019); connectivity between MPFC and PCC strengthens during adolescence (Fan et al. 2021). Acute stressor of challenging math calculation, on the other hand (relative to the subsequent social feedback) evoked bilateral AI activation. Insula is involved in responses to salience, homeostatic incongruence, and cognitive challenge (Gasquoine 2014). The AI is sensitive to the salience of the self‐relevance of feedback, rather than to social feedback itself (Perini et al. 2018). In line with this, we find that insula activation during evaluated conditions reflects the cognitively challenging, acute stress phase of the task, rather than the social aspect.

Adolescence is an important period for shifts in self‐concept with high sensitivity to social comparison (Butterfield, Grad‐Freilich, and Silk 2023). Self‐concept is susceptible to becoming more negative in EA (Cole et al. 2001), and teacher/peer approval correlates with self‐esteem during this period (Harter 1990). In our task, participants were exposed to an evaluative comparison to (virtual) peers after a stressful task, and received different (positive or negative) feedbacks after the same cognitive and psychosocial challenge, allowing us to dissociate evaluative from performative sources of stress. We found that positive feedback produced activations compatible with positive emotions, as similar responses in right frontal pole were observed, together with the putamen, in motivation for rewards (Mizuno et al. 2008). Transcranial stimulation of right PFC increases motivation and willingness to exert effort in young adults (Soutschek et al. 2018) whereas lower grey matter volume in this region is reported in depressed adolescents (Shad, Muddasani, and Rao 2012). These data accord with the notion that positive feedback induces long‐term motivation in adults (Burgers et al. 2015) and suggest that positive feedback in stressful conditions may encourage motivation and willingness to go on, unlike negative feedback.

We also found activation of the superior parietal gyrus during positive feedback compared to negative feedback, a region that is part of the dorsal attention network (Corbetta and Shulman 2002). Precentral gyrus was also active during positive feedback, typically linked to both voluntary movement and overt attention (Bhattacharjee et al. 2021). These effects might reflect an increase in attentional processes and accord with the broaden‐and‐build theory of affect‐driven changes in cognition (Fredrickson 2001). This theory states that positive emotions broaden momentary thought–action repertoires, scopes of attention, cognition and action, where one learns from the environment and becomes motivated to explore (Fredrickson 2001; Fredrickson and Branigan 2005). Conversely, according to this theory, negative emotions narrow this scope, giving way to more fixed action tendencies such as fight or flight (Fredrickson and Branigan 2005). In line with this, healthy and anxious youth are quick to focus their gaze and attention to negative social feedback given by peers (Rosen et al. 2019). Overall, differential brain activations seen during positive feedback in our study could point toward more adaptive and beneficial outcomes of stress regulation after exposure to a challenging evaluated situation.

We exposed participants to similar levels of challenge before delivering either positive or negative feedback. Under these conditions, we did not observe any differential impact of negative feedback across the whole group. Contrary to our expectations, negative feedback did not activate frontal or limbic regions involved in emotion regulation, as described in previous studies using negative valanced MIST (Corr et al. 2020; Dedovic et al. 2005; Pruessner et al. 2008). Although behavioral reactivity to negative feedback decreases in EA, reflecting greater cognitive control with development (Dobbelaar et al. 2023); neural responses tend to show higher reactivity to negative feedback or social rejection during this age period, with strong recruitment of insula (Masten et al. 2009). In our paradigm, different test conditions were pseudorandomized, followed by written feedback only (no oral feedback), and separated by brief rest intervals. This might prevent accumulation effects of successive trials, and the preceding stressor phase was not more intense before the negative than the positive feedback, in contrast to some versions of MIST. This might explain the lack of differential increase to negative evaluation as it might align with the adolescents' self‐concept and expectation of doing poorly on the task and thus evoke no distinctive responses after negative feedback.

Remarkably, a differential effect of negative feedback was observed in the subgroup with higher depressive symptoms. Despite the lesser saliency of negative feedback, compared to the original MIST, participants with mild to moderate depressive scores showed selectively higher activation in the PCC, as well as in the left frontal cortex and cerebellum, when receiving negative feedback. Cortical midline structures implicated in self‐processing such as PCC are known to have an important role in the development of major depressive disorder (for review Butterfield, Grad‐Freilich, and Silk 2023). Healthy adolescents deactivate self‐referential regions such as PCC and precuneus, in response to negative social stimuli, and activate more extensively during positive stimuli that relates to their self‐concept (Butterfield, Grad‐Freilich, and Silk 2023). In line with this, negative feedback might enhance self‐related processing after challenging task conditions in the more vulnerable, though clinically healthy, adolescents.

The age range we chose encapsulating the average age for puberty is a time of immense change for both boys and girls (Brix et al. 2019). It is further complicated by puberty stage and inter‐individual variability. We did not do any analysis regarding sex differences due to the small sample size, especially with the group split according to depressive symptoms. We know that there are sex‐dependent differences in brain white matter and stress response in this group (Schilliger et al. 2024). Regarding feedback, it is quite possible that there will be differences in how boys and girls respond. They might be exposed to different types of stressors due to the changes in their physiology, social interactions, and accordingly their shaping of coping mechanisms (Rew et al. 2012). However, an investigation with larger samples, and additional information on pubertal stage and menstrual cycle is needed to answer this specific question.

## 5 Conclusions

In sum, during EA, negative feedback might enhance the tendency for increased self‐referential processing, especially in vulnerable individuals reporting subclinical depressive symptoms. On the other hand, positive feedback after an acute stress might recruit brain regions linked to positive experience, motivation, and attention, which could encourage healthy development and learning. We suggest that more research should be focused on positive feedback, especially in stress‐related contexts, which could shed new light on the regulation of psychosocial stress and, in turn, modify evaluative processes in a way that could benefit and protect this vulnerable period of neural and mental development.

## Author Contributions

**Zeynep Celen**: investigation, writing–original draft, methodology, visualization, formal analysis, conceptualization, writing–review and editing. **Ryan J. Murray**: writing–review and editing, methodology, investigation. **Mariana Magnus Smith**: project administration, writing–review and editing, investigation. **Sondes Jouabli**: writing–review and editing, investigation. **Vladimira Ivanova**: investigation, writing–review and editing. **Eleonore Pham**: investigation, writing–review and editing. **Zoe Schilliger**: investigation, writing–review and editing, formal analysis. **Patrik Vuilleumier**: writing–review and editing, supervision, methodology. **Arnaud Merglen**: funding acquisition, writing–review and editing, conceptualization. **Paul Klauser**: writing–review and editing, funding acquisition, conceptualization. **Camille Piguet**: conceptualization, funding acquisition, writing–review and editing, supervision.

## Conflicts of Interest

The authors declare no conflicts of interest.

### Peer Review

The peer review history for this article is available at https://publons.com/publon/10.1002/brb3.70154.

## Supporting information



Supporting information

## Data Availability

The data that support the findings of this study are available from the corresponding author upon reasonable request.
