# Supplementary material for: Brain Reactivity and Vulnerability to Social Feedback Following Acute Stress in Early Adolescence
Source: Brain Behav. 2024 Dec 4;14(12):e70154. doi: 10.1002/brb3.70154 (PMC11617326; doi:10.1002/brb3.70154)
Supplement: Supplementary file 1 — Supporting information [file BRB3-14-e70154-s001.docx]

# Supplementary Material

Brain reactivity and vulnerability to social feedback following acute stress in early adolescence.

Zeynep Celen^1,2*^, Ryan J. Murray^1^, Mariana Magnus Smith*^3^*, Sondes Jouabli*^1^*, Vladimira Ivanova*^1^*, Eleonore Pham*^1^*, Zoe Schilliger^4,5^, Patrik Vuilleumier^2^, Arnaud Merglen^3^, Paul Klauser^4,5^, Camille Piguet^1,3^

*^1^Department of Psychiatry, Faculty of Medicine, University of Geneva, Switzerland*

*^2^Department of Neurosciences, Faculty of Medicine, University of Geneva, Switzerland*

*^3^Division of General Pediatrics, Geneva University Hospitals & Faculty of Medicine, University of Geneva, Switzerland*

*^4^Centre for Psychiatric Neuroscience, Department of Psychiatry, Lausanne University Hospital and the University of Lausanne, Lausanne, Switzerland*

*^5^Service of Child and Adolescent Psychiatry, Department of Psychiatry, Lausanne University Hospital and the University of Lausanne, Lausanne, Switzerland*

*Corresponding Author:

Zeynep Celen

Address: Campus Biotech, Chemin des Mines 9, 1202 Geneva, Switzerland.

## **S1. Supplementary Material Methods**

###

### **S1.1 Incongruence of Feedback Exclusive Mask**

In order to decrease the sense of incongruency between the performance and the pre-determined feedback, we simplified the feedback screen compared to previous studies (Murray et al., 2021, 2022). Additionally, we took further precaution to account for a possible ‘feeling’ of incongruence by creating an exclusive incongruence mask. We flagged incongruence as having a positive feedback equal to or below 40% of correct answers or having a negative feedback equal to or above 60% correct answers (Murray et al., 2021, 2022). Congruence was the reverse (negative feedback to <40% and positive feedback to >60%). The binary mask was created using MarsBaR (Brett et al, 2002) from the second level with a cluster forming threshold at p<.001 and corrected at the cluster level FWE p<.05.

##

### **S1.2. fMRI data acquisition and preprocessing**

A 3T Magnetom TIM Trio scanner (Siemens, Germany) with a 32-channel head coil was used to acquire the functional images. Participants used earplugs to protect hearing and foam pads were used to minimize head motion. We recorded MEMPRAGE sequence for the anatomical image, (4 echos, 176 sagital slices TR=2530ms, TE1/TE2/TE3/TE4=1.64/3.5/5.36/7.22ms, slice thickness= 1mm, FOV= 256x240mm, voxel size= 1x1mm, TI=1100ms, flip angle (FA)= 7 degrees,). We used standard echoplanar imaging sequence for fMRI task (2 runs, each run approximately 13 minutes), TR= 2100ms, echo time (TE)= 30ms, 36 transverse slices, FOV= 384x384mm2, voxel size= 3.2mm x 3.2mm x 3.2mm, flip angle (FA)= 80 degrees, 64x64 base resolution). MRI data was collected at the Brain and Behaviour Laboratory (BBL) at the Department of Medicine, University of Geneva, Switzerland.

SPM 12 (SPM12, Wellcome Trust Centre for Neuroimaging, London, United Kingdom) standard procedures were used on Matlab 2021a (Mathworks Inc., Natick, MA, USA) for image pre-processing. Computations were performed using high performance computing at University of Geneva on the “Baobab” and “Yggdrasil” scientific computing clusters provided by the University.

Functional images were realigned and all images plus mean image was resliced and slice time corrected. The mean image was co-registered with the subject’s MEMPRAGE structural image. Then functional and structural images were normalized. For smoothing, 8mm Gaussian kernel was used.

Due to the young age of our population, there was considerable head motion detected during image processing. Therefore, we calculated Framewise Displacement (Power et al., 2012) and excluded participants with more than 20% of scans with higher displacement than 0.9mm (Siegel et al., 2013). Six subjects were excluded as a result of high motion and one subject had to be excluded due to technical problems during scanning. Results are reported from the remaining 61 subjects

###

### **S1.3 First-Level Analysis**

A general linear model of individual fMRI data was designed containing the following events: acute stress (calculation), control condition (ctrl), three types of feedback (Positive FB, Negative FB, Control FB), plus three types of rest periods (Positive R, Negative R, Control R). As regressors of non-interest, there was one column for “non-events”, which were intertrial events, such as fixation crosses and information screens. In addition, 24 columns were added with head motion parameters and their derivatives (Friston et al., 1996). We used FAST autocorrelation algorithm of SPM12 to account for temporal autocorrelation (Olszowy et al., 2019) and a high pass filter of 200Hz.

### **S1.4 Group Level Analysis Details**

#### Test Condition vs Control Condition

We used one-way within-subject ANOVA, with 5 conditions: Acute Stress, Control Condition, Positive FB, Negative FB, Control FB. We added two regressors of no interest: sex, and the percentage of correct answers in the evaluated condition (accuracy) due to the wide range in performance across participants (27.5% to 95%). We compared (Acute Stress + Positive FB+ Negative FB> Control Condition + Control FB) for activation and (Control Condition + Control FB> Acute Stress + Positive FB + Negative FB) for deactivation during the experimental condition.

#### Acute Stress vs Social Feedback

We further compared conditions by examining the specific effects of acute stress (Acute Stress> Positive FB + Negative FB) and those of receiving social feedback (Positive FB + Negative FB >Acute Stress). In order to double check that we capture the regions related to both types of valence, we did a conjunction analysis of (Positive FB > Acute Stress) and (Negative FB > Acute Stress) to investigate the social stress compared to acute stress. Another conjunction analysis was performed to compare acute stress with social feedback, (Acute Stress > Positive FB) and (Acute Stress>Negative FB).

#### Feedback Valence

We performed a one-way within-subject ANOVA with 3 conditions: Positive FB, Negative FB, Control FB and compared valence of feedback (Positive FB > Negative FB) and (Negative FB > Positive FB). Sex and accuracy were added as regressors of non-interest. To minimize any effect due to a sense of incongruency of the subject’s performance with the pre-determined feedback, we applied an exclusive incongruency mask as in Murray et al. (see Supplementary Material).

*Whole Brain Correlations of Clinical Scores*

We investigated the correlation of individual BDI, STAI-C trait, STAI-C state clinical scores with activity in the (Negative FB > Positive FB) contrast using one group t-test on SPM 12.

*BDI subgroup analysis*

The subgroup comparison (depressive symptoms vs no symptoms) of brain activity within the contrasts was performed using a two-sample t-test and probing for the interaction of Group and Valence. We also analysed the possible group differences on Positive FB > Acute Stress; Negative FB > Acute Stress, Acute Stress > Positive FB and Acute Stress > Negative FB.

**S2. Supplementary Material Results**

### **S2.1 Incongruency Mask**

Table S1. *Cluster activations and peak coordinates of the incongruency mask.*

|  | |  |  |  | ***MNI Coordinates*** | | |
| --- | --- | --- | --- | --- | --- | --- | --- |
| ***Brain Region*** | ***Side*** | | ***k*** | ***T*** | ***x*** | ***y*** | ***z*** |
|  | |  |  |  |  |  |  |
| ***Match> Mismatch (no clusters)*** | |  |  |  |  |  |  |
|  | |  |  |  |  |  |  |
| ***Mismatch > Match (3 clusters)*** | |  |  |  |  |  |  |
| No label | | R | 2479 | 4.56 | 4 | 1 | 6 |
| Thalamus | | R |  | 4.13 | 3 | -12 | 14 |
| Caudate | | L |  | 3.85 | -7 | 2 | 10 |
|  | |  |  |  |  |  |  |
| Cerebellum Crus I | | R | 2625 | 4.43 | 20 | -70 | -33 |
| Cerebellum Crus I | | R |  | 4.37 | 17 | -80 | -27 |
| Cerebellum Crus II | | R |  | 3.73 | 30 | -73 | -42 |
|  | |  |  |  |  |  |  |
| Posterior Cingulate Gyrus (PCC) | | R | 1898 | 3.98 | 3 | -43 | 19 |
| PCC | | R |  | 3.82 | 5 | -46 | 11 |
| White Matter/ PCC | | L |  | 3.61 | -7 | -40 | 21 |

Note. MNI: Montreal Neurological Institute

####

**S2.2 Physiology**

### **Fig S1.**

#### Heart Rate Change During Recovery Periods Following Control, Positive or Negative Feedback.


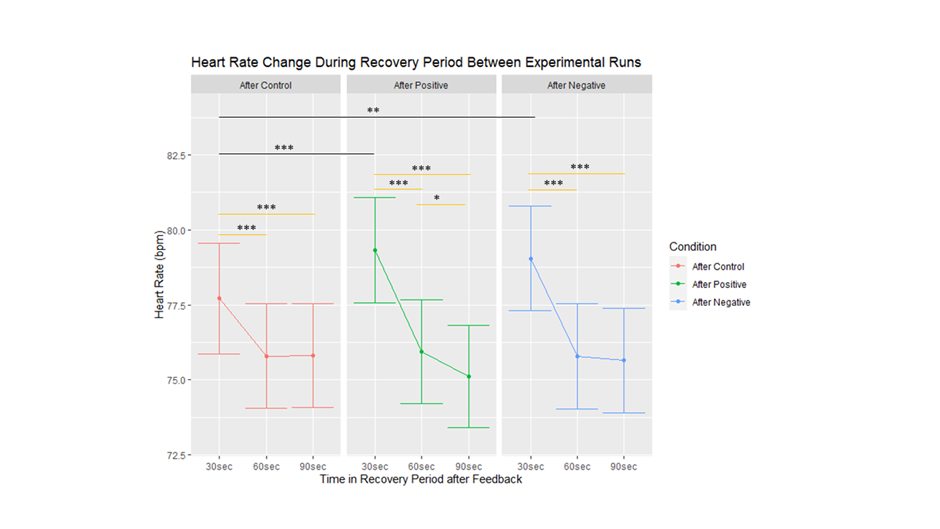


Note. Heart Rate (HR) during recovery period. Mean HR high during the first 30 seconds of the conditions, then there is a decrease till the end of the recovery period in all conditions. Positive R and Negative R had significant higher HR to start with compared to control condition, however were not different than each other. (*** p<.001, **p<.01, * p<.05). Error bars represent standard error. Abbreviations: bpm, beats per minute.

**S2.2.1 BDI-Group Differences in HR**

Two group t-test showed no group diffences in HR (t(1298)=.895, p=.3, M_depsym_= 77.01bpm, M_nosym_=76.46bpm). There were also no interaction of group with condition (positive, negative, control; ConditionXGroup, F(2,3402)= .919, p=.399, η^2^ =0.0005) or group with time point (30, 60, 90; Time Point X Group, F(2,3402)=1.204, p=.3, η^2^ =0.0007).

**S3. Group Level Analysis**

### **Table S2.**

#### Cluster activations and deactivations during Test Condition.

|  |  |  |  |  |  |  |
| --- | --- | --- | --- | --- | --- | --- |
|  |  |  |  | ***MNI Coordinates*** | | |
| ***Brain Region*** | ***Side*** | ***k*** | ***T*** | ***x*** | ***y*** | ***z*** |
|  |  |  |  |  |  |  |
| ***Test > Control (7 clusters)*** | | | | | | |
|  |  |  |  |  |  |  |
| Occipital Pole | R | 55349 | 13.73 | 15 | -98 | 9 |
| Occipital Pole | L |  | 13.6 | -12 | -100 | 7 |
| Occipital Fusiform Gyrus | L |  | 11.4 | -22 | -82 | -14 |
|  |  |  |  |  |  |  |
| No Label | R | 11665 | 6.92 | 28 | 33 | 0 |
| Paracingulate Gyrus | R |  | 6.9 | 8 | 30 | 34 |
| Insula | R |  | 6.31 | 27 | 25 | 0 |
|  |  |  |  |  |  |  |
| Middle Frontal Gyrus | L | 6833 | 6.78 | -42 | 20 | 26 |
| Inferior Frontal Gyrus (opercularis) | L |  | 6.73 | -45 | 11 | 29 |
| Inferior Frontal Gyrus (triangularis) | L |  | 6.24 | -36 | 23 | 21 |
|  |  |  |  |  |  |  |
| Lateral Occipital Cortex/Superior Parietal Lobe | L | 1356 | 5.72 | -26 | -61 | 45 |
| Lateral Occipital Cortex | L |  | 5.16 | -26 | -71 | 36 |
|  |  |  |  |  |  |  |
| Insula | L | 355 | 5.55 | -30 | 22 | -3 |
|  |  |  |  |  |  |  |
| No label | R | 69 | 5.17 | 19 | 48 | -6 |
|  |  |  |  |  |  |  |
| Middle Temporal Gyrus | L | 79 | 5.07 | -52 | -38 | 1 |
|  |  |  |  |  |  |  |
| ***Control > Test (3 clusters)*** |  |  |  |  |  |  |
|  |  |  |  |  |  |  |
| Accumbens | L | 689 | 6.95 | -12 | 5 | -8 |
| Accumbens | R | 304 | 6.08 | 11 | 8 | -6 |
| Supramarginal Gyrus | R | 129 | 5.19 | 62 | -22 | 24 |

Note. One-way Anova within subjects second level analysis (0.5 Acute Stress + 0.25 Positive FB+ 0.25 Negative FB > 0.5 Control Condition + 0.5 Control FB). And the opposite for deactivation. Results are presented with a cluster forming threshold of FWE<.05 and k>10. Abbreviations: MNI: Montreal Neurological Institute.

### **Figure S2.**


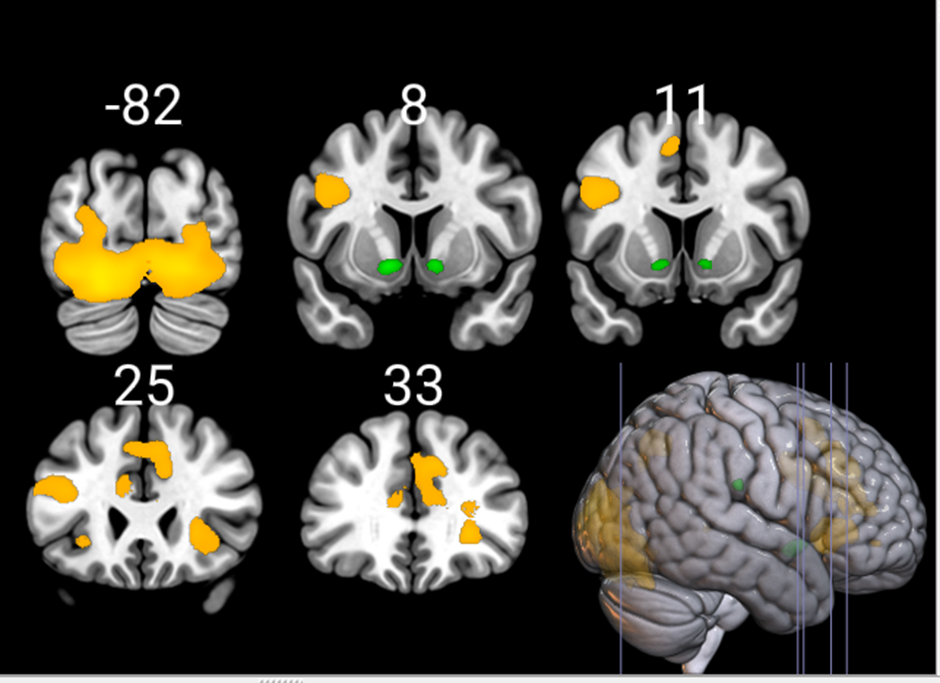


Test condition activations (yellow) and deactivations (green). Cluster forming threshold FWE<.05 with k>10. Values indicate y-axis MNI coordinates.

## **Table S3.**

|  |  |  |  | ***MNI Coordinates*** | | |  |
| --- | --- | --- | --- | --- | --- | --- | --- |
|  |  |  |  |  | | |  |
| ***Brain Region*** | ***Side*** | ***k*** | ***T*** | ***x*** | ***y*** | ***z*** |  |
|  |  |  |  |  |  |  |  |
| ***Feedback > Acute Stress*** |  |  |  |  |  |  |  |
|  |  |  |  |  |  |  |  |
| Posterior Cingulate Gyrus | R | 439721 | 20.06 | 3 | -46 | 32 |  |
| Lateral Occipital Cortex (superior)/Angular Gyrus | L |  | 18.01 | -57 | -61 | 28 |  |
| Lateral Occipital Cortex (superior)/Angular Gyrus | R |  | 17.94 | 56 | -59 | 36 |  |
|  |  |  |  |  |  |  |  |
| Frontal Pole |  | 69774 | 15.21 | 0 | 62 | 13 |  |
| Frontal Medial Cortex/Frontal Pole |  |  | 13.96 | 0 | 53 | -7 |  |
| Frontal Pole | L |  | 13.94 | -5 | 61 | 25 |  |
|  |  |  |  |  |  |  |  |
| Cerebellum Crus I/II | R | 3593 | 9.88 | 24 | -82 | -33 |  |
|  |  |  |  |  |  |  |  |
| Cerebellum IX | R | 2079 | 8.2 | 7 | -50 | -44 |  |
| Cerebellum IX | L |  | 8.13 | -7 | -52 | -44 |  |
|  |  |  |  |  |  |  |  |
| Brainstem |  | 651 | 5.91 | 4 | -23 | -25 |  |
|  |  |  |  |  |  |  |  |
| Postcentral Gyrus | L | 873 | 5.73 | -18 | -31 | 67 |  |
| Postcentral Gyrus | L |  | 5.58 | -22 | -40 | 64 |  |
|  |  |  |  |  |  |  |  |
|  |  |  |  |  |  |  |  |
| ***Acute Stress > Feedback*** |  |  |  |  |  |  |  |
|  |  |  |  |  |  |  |  |
| Lateral Occipital Cortex (superior) | L | 51281 | 12.23 | -18 | -68 | 53 |  |
| Supramarginal Gyrus | L |  | 11.61 | -45 | -40 | 47 |  |
| Superior Frontal Gyrus (dorsolateral) | R |  | 10.97 | 26 | 0 | 56 |  |
|  |  |  |  |  |  |  |  |
| Lateral Occipital Cortex (superior) | R | 13610 | 11.27 | 19 | -65 | 54 |  |
| Superior Parietal Lobule | R |  | 8.64 | 31 | -47 | 48 |  |
| Superior Parietal Lobule | R |  | 8.19 | 31 | -52 | 57 |  |
|  |  |  |  |  |  |  |  |
| Precentral Gyrus | L | 3566 | 10.49 | -47 | 4 | 31 |  |
|  |  |  |  |  |  |  |  |
| Insula | R | 7462 | 9.71 | 32 | 21 | 8 |  |
| No Label |  |  | 6.87 | 22 | 45 | -5 |  |
| Caudate/no label | R |  | 6.06 | 20 | 29 | 5 |  |
|  |  |  |  |  |  |  |  |
| Precentral Gyrus | R | 1602 | 7.74 | 47 | 6 | 30 |  |
|  |  |  |  |  |  |  |  |
| Insula | L | 4576 | 7.7 | -29 | 19 | 10 |  |
| Caudate/no label | L |  | 7.13 | -18 | -3 | 27 |  |
| No label | L |  | 6.39 | -21 | 30 | 9 |  |
|  |  |  |  |  |  |  |  |
| Middle Frontal Gyrus | L | 1864 | 7.26 | -44 | 31 | 26 | |
|  |  |  |  |  |  |  |  |
| Caudate | R | 1017 | 6.62 | 18 | -4 | 28 |  |
|  |  |  |  |  |  |  |  |
| Caudate | R |  | 5.48 | 18 | 9 | 25 |  |
| No label |  |  | 5.25 | 21 | -19 | 27 |  |
|  |  |  |  |  |  |  |  |
| Inferior Temporal Gyrus | L | 233 | 6.04 | -54 | -54 | -13 |  |
|  |  |  |  |  |  |  |  |
| Cerebellum V | R | 182 | 5.7 | 2 | -61 | -17 |  |
|  |  |  |  |  |  |  |  |
| Lateral Occipital Cortex (inferior) | R | 160 | 5.68 | 41 | -65 | 6 |  |
|  |  |  |  |  |  |  |  |
| Vermis VIIIa |  | 194 | 5.66 | 2 | -62 | -34 |  |
|  |  |  |  |  |  |  |  |
| Cerebellum VIIb | R | 16 | 5.44 | 23 | -71 | -47 |  |
|  |  |  |  |  |  |  |  |
| Cerebellum X | L | 12 | 5.41 | -26 | -36 | -42 |  |
|  |  |  |  |  |  |  |  |
| No label | L | 71 | 5.29 | -4 | 20 | 7 |  |
|  |  |  |  |  |  |  |  |
| No label | L | 24 | 5.05 | -4 | 1 | 25 |  |
|  |  |  |  |  |  |  |  |

## S3a. Acute Stress and Social Feedback Conjunction Analysis in the Test Condition.

|  |  |  |  | ***MNI Coordinates*** | | |
| --- | --- | --- | --- | --- | --- | --- |
|  |  |  |  |  | | |
| ***Brain Region*** | ***Side*** | ***k*** | ***T*** | ***x*** | ***y*** | ***z*** |
|  |  |  |  |  |  |  |
| ***Feedback > Acute Stress (Positive FB>Acute Stress Λ Negative FB>Acute Stress)*** | | | | | | |
|  | | | | | | |
| Posterior Cingulate Gyrus | R | 216026 | 16.07 | 3 | -46 | 31 |
| Lateral Occipital Cortex (superior)/Angular Gyrus | R |  | 14.07 | 58 | -59 | 34 |
| Occipital Pole | R |  | 14.02 | 11 | -88 | 35 |
|  |  |  |  |  |  |  |
| Lateral Occipital Cortex (superior)/Angular Gyrus | L | 82831 | 14.5 | -57 | -61 | 28 |
| Middle Temporal Gyrus | L |  | 12.95 | -60 | -16 | -9 |
| Lateral Occipital Cortex (superior) | L |  | 12.48 | -52 | -68 | 34 |
|  |  |  |  |  |  |  |
| Frontal Pole | R | 46796 | 12.14 | 1 | 61 | 21 |
| Frontal Pole | R |  | 11.39 | 12 | 60 | 30 |
| Frontal Pole | R |  | 11.15 | 0 | 56 | 0 |
|  |  |  |  |  |  |  |
| Left Crus I | L | 2935 | 9.19 | -22 | -82 | -33 |
|  |  |  |  |  |  |  |
| Right Crus II | R | 2514 | 8.13 | 23 | -82 | -35 |
|  |  |  |  |  |  |  |
| Precentral Gyrus | R | 2827 | 8.13 | 37 | -17 | 43 |
|  |  |  |  |  |  |  |
| Inferior Frontal Gyrus, pars triangularis | R | 3186 | 7.4 | 53 | 30 | 5 |
| Frontal Orbital Cortex/Frontal Pole | R |  | 7 | 37 | 33 | -13 |
| Frontal Pole | R |  | 6.87 | 48 | 35 | -10 |
|  |  |  |  |  |  |  |
| Middle Frontal Gyrus | R | 1131 | 6.79 | 44 | 20 | 46 |
|  |  |  |  |  |  |  |
| Postcentral Gyrus | R | 1538 | 6.45 | 27 | -28 | 63 |
| Superior Parietal Lobule | R |  | 5.5 | 23 | -42 | 64 |
|  |  |  |  |  |  |  |
| Brainstem | R | 303 | 6.33 | 7 | -50 | -44 |
|  |  |  |  |  |  |  |
| Occipital Pole | L | 301 | 6.17 | -20 | -102 | 0 |
|  |  |  |  |  |  |  |
| Lateral Occipital Cortex (inferior) | L | 305 | 5.88 | -41 | -88 | 11 |
| Lateral Occipital Cortex (inferior) | L |  | 5.48 | -43 | -87 | -2 |
|  |  |  |  |  |  |  |
| Left IX | L | 207 | 5.74 | -6 | -53 | -46 |
|  |  |  |  |  |  |  |
| Occipital Fusiform Gyrus | R | 184 | 5.43 | 31 | -74 | -10 |
| Ventricle | R | 316 | 5.42 | 5 | -1 | 13 |
| Ventricle | L |  | 5.16 | -5 | 5 | 11 |
|  |  |  |  |  |  |  |
| Thalamus | L | 63 | 5.38 | -12 | -30 | 1 |
|  |  |  |  |  |  |  |
| Pre/Postcentral Gyrus | L | 47 | 5.25 | -37 | -18 | 38 |
|  |  |  |  |  |  |  |
| Middle Frontal Gyrus | L | 192 | 5.23 | -36 | 15 | 44 |
|  |  |  |  |  |  |  |
| Supplementary motor cortex | R | 19 | 5.04 | 7 | -14 | 62 |
|  |  |  |  |  |  |  |
| Occipital Fusiform Gyrus | L | 41 | 5 | -20 | -85 | -19 |
|  |  |  |  |  |  |  |
| Postcentral Gyrus | R | 106 | 4.98 | 59 | -7 | 38 |
|  |  |  |  |  |  |  |
|  |  |  |  |  |  |  |
| ***Acute Stress > Feedback (Acute Stress > Positive FB Λ Acute Stress> Negative FB)*** | | | | | | |
|  |  |  |  |  |  |  |
|  |  |  |  |  |  |  |
| Supramarginal Gyrus/Superior Parietal Lobule | L | 27965 | 9.76 | -44 | -40 | 47 |
| Lateral Occipital Cortex (Superior) | L |  | 9.33 | -19 | -66 | 56 |
| Postcentral Gyrus | L |  | 9.19 | -47 | -33 | 51 |
|  |  |  |  |  |  |  |
| Lateral Occipital Cortex (Superior) | R | 4902 | 8.51 | 19 | -64 | 54 |
| Superior Parietal Lobule | R |  | 6.59 | 31 | -47 | 48 |
| Superior Parietal Lobule | R |  | 6.13 | 31 | -52 | 57 |
|  |  |  |  |  |  |  |
| Precentral Gyrus | L | 1951 | 8.43 | -47 | 5 | 30 |
|  |  |  |  |  |  |  |
| Frontal Operculum/Insula | R | 1626 | 8.2 | 33 | 20 | 9 |
|  |  |  |  |  |  |  |
| Superior Frontal Gyrus / Middle Frontal Gyrus | R | 2326 | 8.14 | 26 | 0 | 56 |
|  |  |  |  |  |  |  |
| Paracingulate Gyrus | L | 3475 | 7.4 | -3 | 9 | 53 |
| Paracingulate Gyrus | R |  | 6.92 | 8 | 17 | 46 |
|  |  |  |  |  |  |  |
| Precentral Gyrus | R | 497 | 6.28 | 47 | 6 | 30 |
|  |  |  |  |  |  |  |
| Insular Cortex | L | 799 | 6.18 | -29 | 19 | 10 |
| WM | L |  | 5.23 | -21 | 30 | 9 |
| WM | L |  | 5.14 | -20 | 23 | 17 |
|  |  |  |  |  |  |  |
| Middle Frontal Gyrus # dlPFC | L | 345 | 5.52 | -42 | 31 | 25 |
|  |  |  |  |  |  |  |
| WM | R | 161 | 5.48 | 19 | -4 | 28 |
| WM | L | 124 | 5.36 | -18 | -3 | 27 |
|  |  |  |  |  |  |  |
| Supramarginal Gyrus/Postcentral | R | 192 | 5.34 | 48 | -32 | 51 |
| Supramarginal Gyrus | R |  | 4.92 | 43 | -35 | 44 |
|  |  |  |  |  |  |  |
| Inferior Temporal Gyrus | L | 16 | 4.94 | -53 | -51 | -14 |
|  |  |  |  |  |  |  |
|  |  |  |  |  |  |  |

Note. Results presented with a cluster forming threshold of FWE<.05 and k>10. MNI: Montreal Neurological Institute.

**Table S4.**

*Whole brain analysis of valence of social feedback between the two groups according to BDI scores.*

|  |  |  |  | ***MNI Coordinates*** | | |
| --- | --- | --- | --- | --- | --- | --- |
| ***Brain Region*** | ***Side*** | ***k*** | ***T*** | ***x*** | ***y*** | ***z*** |
|  |  |  |  |  |  |  |
| ***Positive FB > Negative FB X Depressive Symptoms > No Symptoms***  ***(no clusters)*** | | | | |  |  |
|  |  |  |  |  |  |  |
| ***Negative FB > Positive FB X Depressive Symptoms > No Symptoms (7 clusters)*** | | | | | | |
| Cerebellum Crus I | L | 1331 | 5.21 | -40 | -66 | -29 |
| Cerebellum VI | L |  | 3.71 | -29 | -67 | -21 |
| Cerebellum Crus I | L |  | 3.52 | -38 | -76 | -26 |
|  |  |  |  |  |  |  |
| Fusiform Cortex | R | 2943 | 4.88 | 36 | -52 | -12 |
| Cerebellum Crus 1 | R |  | 4.23 | 40 | -53 | -31 |
| Cerebellum VI | R |  | 4.16 | 39 | -57 | -23 |
|  |  |  |  |  |  |  |
| Posterior Cingulate Gyrus | L | 6404 | 4.65 | -4 | -28 | 34 |
| Posterior Cingulate Gyrus | L |  | 4.44 | -1 | -35 | 29 |
| Precuneus/Posterior Cingulate | R |  | 4.18 | 14 | -39 | 37 |
|  |  |  |  |  |  |  |
| Cerebellum VIIIa | R | 1959 | 4.44 | 33 | -53 | -47 |
| Cerebellum IX | R |  | 4.32 | 9 | -56 | -46 |
| Cerebellum VIIIb | R |  | 4.15 | 25 | -51 | -47 |
|  |  |  |  |  |  |  |
| Posterior Cingulate Gyrus | L | 1543 | 4.38 | -6 | -42 | 3 |
| Brain Stem | L |  | 4.29 | -1 | -31 | 0 |
| Posterior Cingulate Gyrus | R |  | 3.72 | 9 | -41 | 4 |
|  |  |  |  |  |  |  |
| Frontal Pole | L | 2271 | 4.25 | -26 | 44 | 12 |
| No label | L |  | 4.05 | -10 | 28 | 13 |
| Inferior Frontal Gyrus (Triangularis) | L |  | 3.91 | -40 | 33 | 11 |
|  |  |  |  |  |  |  |
| Cerebellum VI | R | 1709 | 4.06 | 11 | -68 | -15 |
| Cerebellum V | L |  | 4.04 | -6 | -63 | -15 |
| Cerebellum V | R |  | 3.77 | 1 | -60 | -18 |

Note. Two group t-test. All results have a cluster forming threshold of p<.001 and are FWE<.05 corrected at the cluster level. An exclusive incongruence mask is used to account for possible sense of incongruency between performance and the predetermined feedback received. Abbreviations: BDI; Beck’s Depression Inventory.

**S4. Whole Brain Correlations**

There were no significant clusters that correlated with BDI, STAI-C trait and STAI-C state scores that passed our threshold (FWE<.05) with the exclusive incongruency mask in the whole group.
